# Attitudes of dentists and interns in Riyadh to the use of dental amalgam

**DOI:** 10.1186/s13104-016-2294-x

**Published:** 2016-11-17

**Authors:** Fahad Alkhudhairy

**Affiliations:** Department of Restorative Dental Sciences, College of Dentistry, King Saud University, Riyadh, Saudi Arabia

**Keywords:** Amalgam, Dental fillings, Dental materials, Dentists’ opinion

## Abstract

**Background:**

Studies investigating the attitudes of Saudi dentists to the use of amalgam for restorations are relatively rare. Considering the goals set forth by the Minamata Convention on Mercury, it appears prudent to investigate the attitudes of experienced dentists and fresh dental graduates to the use of amalgam. The aim of this study was to assess the attitudes of Saudi dentists and interns working in Riyadh, Saudi Arabia to the use of amalgam. Using a convenience sampling methodology, a total of 400 Saudi dentists and interns were contacted to request their participation in this cross-sectional questionnaire-based study. The questionnaire consisted of socio-demographic and practice characteristics such as gender, type of practice, as well as their service sector and questions related to the use of dental amalgam. The data obtained was analyzed using Chi square tests to compare differences in distribution between groups. P values of less than 0.05 were considered statistically significant.

**Results:**

The overall response rate was 84% (336 of 400 potential participants). The majority of the participants (80.7%) did not use dental amalgam for restorations in their clinical practice frequently. A significantly higher number of participants working in private sector did not use amalgam frequently (P = 0.004), agreed on replacing good amalgam restoration with composite resin (P < 0.000) and on stopping the use of amalgam as a final restoration (P = 0.017) compared to participants working in public sector. A significantly higher number of interns did not use amalgam in their clinical practice frequently (P < 0.000), agreed on replacing good amalgam restoration with composite resin (P = 0.002) and on stopping the use of amalgam as a final restoration (P < 0.000) compared to dentists.

**Conclusions:**

Within the limitations of this study, dental amalgam seems to be less frequently used among the surveyed Saudi dentists and interns working in Riyadh. Fresh dental graduates used amalgam less frequently compared to experienced dentists. Furthermore, private dental practitioners showed a propensity to replace existing well-placed amalgam restorations with resin composite which reinforces their market-oriented attitude reported in earlier studies.

**Electronic supplementary material:**

The online version of this article (doi:10.1186/s13104-016-2294-x) contains supplementary material, which is available to authorized users.

## Background

Amalgam was the material of choice for direct posterior restorations till the 1980s [1]. Studies conducted in the 1990s reported that resin composite was used more commonly to restore posterior teeth compared to amalgam [2, 3]. Factors such as greater emphasis on the preservation of tooth structure [4], new and improved restorative materials [5] and patients’ desire to have more esthetic restorations [6] may have resulted in this change in preferences of dentists and patients. Apart from amalgam being unesthetic, the controversy regarding its use is mostly associated with the fact that the restoration consists of about 50% Mercury [7].

Some government and other organizations are against the use of amalgam whereas others support its use as a restorative material. For example, the Norwegian Ministry of Environment banned the use of amalgam as of 1 January 2008 [8]. On the other hand, the Scientific Committee of the European Commission reported in 2008 that amalgam is effective and none of the available direct restorative materials are free of clinical limitations and/or potential biological side effects [9, 10]. However, in 2013, the Minamata Convention on Mercury which is an international treaty governing the mining, use and trade in Mercury had committed to a worldwide reduction and ultimate elimination in the production and use of mercury containing products [11].

Several studies have investigated various aspects of the use of restorative materials for restoring posterior teeth [1–3, 6, 12–15]. However, studies investigating the attitudes of Saudi dentists to the use of amalgam for restorations are relatively rare. A study in 1995 by Khairuldean and Sadig [16] assessed dental practitioners’ knowledge of amalgam toxicity, attitude to removal of amalgam restorations upon the request of the patients and the opinion on the available alternative materials to amalgam. Considering the goals set forth by the Minamata Convention on Mercury [11], it appears prudent to investigate the attitudes of experienced dentists and fresh dental graduates to the use of amalgam. Therefore, the aim of this study was to assess the attitudes of Saudi dentists and interns working in Riyadh, Saudi Arabia to the use of amalgam.

## Methods

The ethical approval for conducting this cross-sectional study among dentists and interns (those attending a formal compulsory one-year program which provides practical experience for beginners in the dental profession) working in various private and public sectors in Riyadh, Saudi Arabia was obtained from the College of Dentistry Research Center (CDRC Registration Number: IR0118). The survey ensured confidentiality, was voluntary and informed written consent was provided by each subject as per the ethical principles of the World Medical Association Declaration [17]. A 10-item Arabic-language questionnaire was developed and pretested in a group of ten Saudi dentists and interns. Difficulties regarding the comprehension of the questionnaire were identified and addressed according to the results of this pilot study. The final questionnaire (see Additional file 1) consisted of socio-demographic and practice characteristics such as gender, type of practice, as well as their service sector. The dentists were asked about their opinion toward amalgam restorations nowadays, whether or not amalgam is being used frequently in their dental practice and for what type of restorations it is used with options like simple restorations, large restorations and core build up. The survey also asked about the reasons that restrict dental amalgam use, whether amalgam is an occupational risk factor at workplace, their opinion about replacing amalgam with alternative tooth colored restorations such as composite and whether they agree or disagree with stopping the use of amalgam for restorations.

Using a convenience sampling methodology, a total of 400 Saudi dentists and interns (11.1% of dentists working in Riyadh) were contacted to request their participation in this study. The dentists’ willingness to participate in the study was sought by the co-investigators and the questionnaires were given to those who agreed to participate to be completed and returned immediately during the period from September 2015 to February 2016. The responses of the participants were entered electronically into the SPSS for Windows version 20 (SPSS Inc., Chicago, IL, USA). The data obtained was analyzed using Chi square tests to compare differences in distribution between groups. P values of less than 0.05 were considered statistically significant.

## Results

The overall response rate was 84% (336 of 400 potential participants). The demographic characteristics of the study population are presented in Table 1.

**Table 1:** Demographic characteristics of the study population

Variables	Total n (%)
Gender^a^	
Male	162 (48.5)
Female	172 (51.5)
Service sector^b^	
Private	178 (55.3)
Public	144 (44.7)
Type of practice^c^	
Interns	213 (63.6)
General practitioners	64 (19.1)
Specialists	27 (8.1)
Consultants	31 (9.3)

Missing values

^a^2

^b^14

^c^1

The overall percentage distribution of the responses to the questionnaire regarding the use of amalgam and according to gender is given in Table 2. The majority of the participants (80.7%) did not use dental amalgam for restorations in their clinical practice frequently. About 77% of the participants reported that esthetics was the major reason for restricting the use of dental amalgam. About 62% of the participants reported that amalgam is not an occupational risk factor at their workplace. Furthermore, the majority of the participants disagreed on replacing good amalgam restoration with composite resin (72.3%) and on stopping the use of amalgam as a final restoration (58%).

**Table 2:** The overall percentage responses to questions related to use of dental amalgam and according to gender

	Male	Female	P value	Overall
Do you use dental amalgam for restorations in your clinical practice frequently?				
Yes	46 (29.1)	17 (9.9)	0.000^a^	64 (19.3)
No	112 (70.9)	154 (90.1)		267 (80.7)
Do you use dental amalgam for the following?^b^				
Simple restorations	20 (12.3)	9 (5.2)	–	29 (8.6)
Large restorations	79 (48.8)	86 (50.0)		166 (49.4)
Build up material	53 (32.7)	52 (30.2)		106 (31.5)
Core material	50 (30.9)	33 (19.2)		85 (25.3)
Not used	47 (29.0)	61 (35.5)		108 (32.1)
What are the reasons that restrict you from using dental amalgam?^b^				
Esthetics	118 (72.8)	139 (80.8)	–	259 (77.1)
Mercury toxicity	36 (22.2)	53 (30.8)		90 (26.8)
Patient’s desire	96 (59.3)	99 (57.6)		197 (58.6)
Other reasons	17 (10.5)	8 (4.7)		25 (7.4)
Is dental amalgam an occupational risk factor at your workplace?				
Yes	42 (26.1)	85 (49.7)	0.000^a^	127 (37.8)
No	119 (73.9)	86 (50.3)		207 (61.6)
Do you agree or disagree on replacing good amalgam restoration with composite resin?				
Agree	29 (18.1)	59 (34.5)	0.001^a^	90 (26.8)
Disagree	131 (81.9)	112 (65.5)		243 (72.3)
If a patient had defective amalgam restoration, what would you prefer changing it to?				
Amalgam	121 (79.6)	146 (86.4)	–	269 (80.1)
Composite	27 (17.8)	21 (12.4)		48 (14.3)
Any of the above	4 (2.6)	2 (1.2)		6 (1.8)
Do you agree or disagree on stopping the use of amalgam as a final restoration?				
Agree	42 (26.1)	95 (55.6)	0.000^a^	139 (41.4)
Disagree	119 (73.9)	76 (44.4)		195 (58.0)

^a^Statistically significant

^b^Multiple responses possible

A significantly higher number of female participants did not use amalgam in their clinical practice frequently compared to male participants (P < 0.000) whereas a significantly higher number of male participants reported that amalgam is not an occupational risk factor at their workplace compared to female participants (P < 0.001). Moreover, a significantly higher number of male participants disagreed on replacing good amalgam restoration with composite resin (P = 0.001) and on stopping the use of amalgam as a final restoration (P < 0.000) compared to female participants.

A significantly higher number of participants working in private sector did not use amalgam frequently (P = 0.004), agreed on replacing good amalgam restoration with composite resin (P < 0.000) and on stopping the use of amalgam as a final restoration (P = 0.017) compared to participants working in public sector. On the other hand, a significantly higher number of participants working in public sector reported that amalgam is not an occupational risk factor at their workplace compared to participants working in private sector (P = 0.001). Esthetics was the reason for the majority of respondents in public (n = 114; 79.2%) and private (n = 136; 76.4%) sectors for restricting the use of dental amalgam. However, if a patient had defective amalgam restoration, the majority of respondents in public (n = 111; 80.4%) and private (n = 145; 84.8%) preferred replacing it with dental amalgam rather than composite material.

A significantly higher number of interns did not use amalgam in their clinical practice frequently (P < 0.000), agreed on replacing good amalgam restoration with composite resin (P = 0.002) and on stopping the use of amalgam as a final restoration (P < 0.000) compared to dentists. On the other hand, a significantly higher number of dentists reported that amalgam is not an occupational risk factor at their workplace compared to interns (P < 0.001). Esthetics was the reason for the majority of interns (n = 162; 76.1%) and dentists (n = 96; 78.7%) for restricting the use of dental amalgam. However, if a patient had defective amalgam restoration, the majority of interns (n = 187; 87.8%) and dentists (n = 81; 74.3%) preferred replacing it with dental amalgam rather than composite material.

## Discussion

Several aspects are to be considered while discussing the use of dental materials including (a) the chemical and biologic properties of the materials and (b) the attitudes and beliefs of dentists and patients [15]. The restoration of posterior teeth using resin composite has been increasingly taught in dental schools worldwide [18–23]. Furthermore, amongst several measures mentioned in the text and annexes of the Minamata Convention on Mercury, it also states important measures such as setting national objectives aiming at minimizing use of dental amalgam, promoting use of cost-effective and clinically effective mercury-free alternatives for dental restoration, restricting the use of dental amalgam to its encapsulated form, promoting the use of best environmental practices in dental facilities to reduce releases of mercury and mercury compounds to water and land, and encouraging representative professional organizations and dental schools to educate and train dental professionals and students on the use of mercury-free dental restoration alternatives and on promoting best management practices [11]. At the backdrop of this information, the aim of the present study was to assess the attitudes of dentists and interns in Riyadh, Saudi Arabia regarding the use of dental amalgam. A comparative assessment of the attitudes of dentists and fresh dental graduates (interns) will give a broader perspective of the differences in attitudes.

The majority of the participants in this study (80.7%) reported that they do not use dental amalgam frequently in their clinical practice which is consistent with the results of previous studies which found that amalgam was used less frequently compared to resin composite [2, 3]. Among those who use dental amalgam frequently, it is noteworthy that a significantly higher number of participants working in public sector compared to those working in private sector used amalgam (P = 0.004). Furthermore, a significantly higher number of participants working in private sector agreed on replacing good amalgam restoration with resin composite compared to those working in public sector (P < 0.001). These results concur with those of previous studies [15, 16] and may be due to the fact that private practitioners are more market-oriented, as elucidated by the authors of these studies. A significantly higher number of fresh dental graduates reported that they were not using dental amalgam frequently compared to experienced dentists (P < 0.001). This may indicate a positive influence of the attributes of the Minamata Convention on Mercury [11] on the dental curriculum regarding the use of dental amalgam.

The majority of the participants in this study (61.6%), a significantly higher number of male participants and those working in public sector reported that amalgam is not an occupational risk factor at their workplace which is contrary to the results of a study among Nordic dentists [15]. Only a small proportion of Nordic dentists were worried about amalgam as an occupational risk factor. Furthermore, sex or working sector of the Nordic dentists did not affect this view.

Dentists may utilize a variety of sources to support decisions in clinical practice which may vary depending on the years since graduation and between general practitioners and specialists [24]. A significantly higher number of dentists used amalgam frequently compared to fresh dental graduates in the present study (P < 0.001). Restorative decision-making is complex and is influenced by several factors. Dentists reportedly are more likely to prefer amalgam for patients who exhibit high caries experience [12, 14] or if the extent of caries is high [14]. Large restorations (49.4%) followed by crown build-up (31.5%) were the most common restorative options for which the participants of this study preferred using amalgam.

Esthetics (77.1%) followed by patients’ desire (58.6%) were reported as the most common reasons to restrict the use of amalgam by the participants of this study. Vidnes-Kopperud et al. [14] reported that the participants of their study preferred to use tooth-colored restorative materials in areas of the mouth that are visible. The authors also concluded that the participants considered esthetics as more important for females. Furthermore, Espelid et al. [13] reported that esthetics were of major concern for patients over longevity of the restorations irrespective of gender. About 27% of the participants of this study reported Mercury toxicity as a reason for restricting the use of amalgam. A survey conducted in Saudi Arabia in 1995 [16] reported that the majority of the dentists (85%) believed that amalgam is safe for patients but 88% indicated that it is hazardous to the dentists if not handled properly.

About 72% of the participants of this study disagreed on replacing good amalgam restorations with resin composite. On the other hand, the survey by Khairuldean and Sadig [16] reported that 63% of their respondents would explain their beliefs to the patients before removing amalgam, 21% would remove amalgam restoration upon patients’ request, 14% would not comply with the patients’ request whereas, 6% would encourage the patients to replace amalgam restoration with an alternative restorative material. A study comparing the mercury levels in general dentists with that in other health professionals using toenail clippings as a biomarker concluded that the number of dental amalgams was not related to the level of toenail mercury levels among dentists, dental specialists and patients. However, the toenail mercury levels of general dentists were found to be more than twice that of non-dental health professionals. The authors also suggested that the avoidance of amalgam cannot be justified by the presence of mercury released from dental amalgam [25].

Amalgam was reported as the most common restorative material of choice (80.1%) in case of changing an existing defective amalgam restoration by the participants of this study. This may be due to the benefits of amalgam perceived by dentists such as less risk of secondary caries compared to tooth colored alternatives [26] or due to operator skills or technique sensitivity involved in restoring with resin composites [5, 27].

The limitations of this study should be considered when interpreting the results. The sampling methodology and cross-sectional study design implemented in this study are major limitations. All dentists working in Riyadh may not be sufficiently represented while using a convenience sample. Although we intended to assess the attitudes of Saudi dentists and interns regarding the use of amalgam, inclusion of non-Saudi dentists would have made the study sample more representative of the dentist population working in Riyadh. A comparative assessment of all aspects of our results with that of similar previous studies [15, 16] was not always possible due to differences in questions and answer choices.

## Conclusions

Within the limitations of this study, dental amalgam seems to be less frequently used among the surveyed Saudi dentists and interns working in Riyadh. Fresh dental graduates used amalgam less frequently compared to experienced dentists. Esthetics and patients’ desire were found to the major reasons for restricting the use of amalgam among the participants. Furthermore, private dental practitioners showed a propensity to replace existing well-placed amalgam restorations with resin composite which reinforces their market-oriented attitude reported in earlier studies.

## Additional files

**Additional file 1.** Questionnaire.

**Additional file 2.** Survey data.

## Abbreviations

CDRC: College of Dentistry Research Center
SPSS: statistical package for the social sciences
