# Supplementary material for: Attitudes of dentists and interns in Riyadh to the use of dental amalgam
Source: BMC Res Notes. 2016 Nov 17;9:488. doi: 10.1186/s13104-016-2294-x (PMC5114813; doi:10.1186/s13104-016-2294-x)
Supplement: Supplementary file 1 — Additional file 1. Questionnaire. [file 13104_2016_2294_MOESM1_ESM.docx]

Questionnaire

1. Gender: Male Female
2. Service sector: Public Private
3. Type of practice: Intern General practitioner Specialist

Consultant

1. Do you use dental amalgam for restorations in your clinical practice frequently?

Yes No

1. Do you use dental amalgam for the following?

Simple restorations Large restorations Build up material Core material Not used

1. What are the reasons that restrict you from using dental amalgam?

Esthetics Mercury toxicity Patient’s desire

Other reasons Specify:…………………………………………………………………………………….

1. Is dental amalgam an occupational risk factor at your workplace?

Yes No

1. Do you agree or disagree on replacing good amalgam restoration with composite resin?

Agree Disagree

1. If a patient had defective amalgam restoration, what would you prefer changing it to?

Amalgam Composite Any of them

1. Do you agree or disagree on stopping the use of amalgam as a final restoration?

Agree Disagree
