# Supplementary material for: Attitudes of dentists and interns in Riyadh to the use of dental amalgam
Source: BMC Res Notes. 2016 Nov 17;9:488. doi: 10.1186/s13104-016-2294-x (PMC5114813; doi:10.1186/s13104-016-2294-x)
Supplement: Supplementary file 2 — Additional file 2. Survey data. [file 13104_2016_2294_MOESM2_ESM.pdf]

Sn	Sample	D_1	D_2	D_3	Q_1	Q_2a	Q_2b	Q_2c
40	2	2	1	1	2	2	2	2
41	2	2	1	1	2	2	1	1
42	2	2	1	4	2	2	1	2
43	2	1	1	3	2	2	2	1
44	2	2	1	1	2	2	1	2
45	2	2	1	2	1	1	1	1
46	2	1	2	2		2	1	2
47	2	1	2	2	2	2	1	2
48	2	1	2	3	2	2	2	2
49	2	1	2	3	2	2	2	1
50	2	1	1	3	1	1	1	1
51	2	1	2	2	2	2	1	1
52	2	2	2	2	1	1	1	1
53	2	2	2	2	2	2	2	2
54	2	1	2	2	1	2	1	2
55	2	1	2	2	2	1	2	2
56	2	1	2	2	2	2	2	2
57	2	1	2	2	2	2	2	2
58	2	1	2	1	2	2	2	2
59	2	1	2	1	2	2	2	2
60	2	1	2	1	2	2	2	2
61	2	1	1	4		1	2	2
62	2	1	1	4		1	2	2
63	2	1	1	4	2	2	2	2
64	2	1	1	4	2	2	2	2
65	2	1	2	2	2	2	2	2
66	2	1	2	2	2	2	2	2
67	2	1	1	4	2	2	1	2
68	2	1	1	4	2	2	1	2
69	2	1	2	4	2	2	2	2
70	2	1	2	4	2	2	2	2
71	2	1	1	3	2	2	1	1
72	2	1	1	3	2	2	1	1
73	2	1	1	4	2	2	2	2
74	2	1	1	4	2	2	2	2
75	2	2	1	1	2	2	1	2
76	2	2	1	1	2	2	1	1
77	2	2	1	1	1	2	1	1
78	2	2	2	2	2	2	2	2
79	2	1	2	2	2	1	2	2
80	2	1	2	3	2	2	2	2
81	2	1	1	4	1	2	2	1
82	2	1	1	4	1	2	2	1
83	2	1	1	4	2	2	1	1
84	2	1	1	4	2	2	1	1
85	2	1	2	2	1	1	1	2

86	2	2	1	1	1	2	1	1
87	2	2	1	1	2	2	1	2
88	2	1	1	4	2	2	2	2
89	2	1	1	4	2	2	2	1
90	2	1	1	4	2	2	2	1
91	2	1	1	4	1	2	1	1
92	2	2	1	1	2	2	1	1
93	2	2	1	1	2	2	1	1
94	2	2	1	1	1	2	1	2
95	2	2	1	1	2	2	1	2
96	2	2	1	1	2	2	1	2
97	2	1	1	1	2	2	1	1
98	2	2	2	2	1	1	1	2
99	2	1	2	3	1	1	2	2
100	2	1	1	2	1	2	1	1
101	2	1	2	3	2	2	2	2
102	2	2	1	1	1	2	1	2
103	2	2	1	1	1	2	1	2
104	2	1	2	3	1	2	1	2
105	2	2	2	3	1	2	1	1
106	2	1	1	2	2	2	2	2
107	2	2	1	1	2	2	1	2
108	2	1	2	3	2	1	1	1
109	2	1	2	2	2	2	1	2
110	2	2	2	3	1	2	1	1
111	2	1	2	2	2	2	2	2
112	2	1	1	2	2	2	2	2
113	2	1	2	2	2	2	1	2
114	2	2	2	2	2	2	2	2
115	2	1	1	3	2	2	2	2
116	2	2	1	3	2	2	2	1
117	2	1	2	2	2	2	2	2
118	2	1	1	2	1	1	1	2
119	2	2	1	1	2	2	1	1
120	2	2	1	1	2	2	1	1
121	2	2	1	1	2	2	1	2
122	2	2	1	4	2	2	1	2
123	2	2	1	4	2	2	1	2
124	2	2	1	1	2	2	1	2
125	2	1	2	2	2	2	2	2
126	2	2	2	2	2	2	1	1
127	2	1	1	1	2	2	1	1
128	2	1	1	1	2	1	2	2
129	2	1	1	1	2	2	2	2
130	2	1	1	1	2	2	2	2
131	2	1	2	1	2	2	1	1
132	2	1	1	4	1	2	1	1

133	2	2	1	1	2	1	2	2
134	2	2	1	2	1	1	1	1
135	2	1	1	1	1	2	1	1
136	2	2	1	1	2	2	1	1
137	2	1	1	3	2	2	2	1
138	2	1	1	4	1	1	2	2
139	2	1	1	1	2	2	2	2
140	2	1	1	4	2	2	2	2
141	2	1	1	4	2	2	2	2
142	2	1	1	3	1	1	1	2
143	2	1	1	1	2	2	1	2
144	2	1	1	2	2	2	2	2
145	2	2	2	3	2	2	2	2
147	2	1	2	2	1	2	1	2
148	2	1	1	1	1	2	1	1
149	2	1	1	1	2	2	2	2
150	2	1	1	3	2	2	2	2
151	2	1	1	2	2	2	2	2
152	2	2	2	2	2	2	2	2
153	2	1	2	2	1	2	1	2
154	2	1	2	2	2	2	2	2
155	2	2	2	2	2	2	2	2
156	2	2	2	2	2	2	2	2
157	2	1	2	3	1	1	1	2
158	2	2	2	2	2	2	1	2
159	2	1	2	4	1	2	1	2
160	2	1	2	2	2	2	2	2
161	2	1	2	2	2	2	2	2
162	2	1	2	3	2	2	1	2
163	2	1	2	2	1	2	1	2
164	2	1	2	2	1	2	1	2
165	2	1	2	2	1	2	1	2
166	2	1	1	1	2	2	2	2
167	2	1	1	1	2	2	1	2
168	2	1	1	1	2	2	1	2
169	2	1	1	1	2	2	2	2
170	2	2	1	1	2	2	1	2
171	2	1	1	1	2	2	1	1
172	2	1	1	2	2	2	2	2
173	2	2	1	1	2	1	2	2
174	2	1	1	1	2	2	1	2
175	2	1	1	1	1	2	1	2
176	2	1	1	1	1	2	1	2
177	2	1	1	1	2	2	1	2
178	2	1	1	1	2	2	1	2
179	2	1	1	1	1	1	2	2
180	2	1	1	1	2	2	1	1

181	2	1	1	1	2	2	2	2
182	2	1	1	1	1	2	1	2
183	2	1	1	1	2	2	1	1
184	2	1	1	1	2	2	2	2
185	2	1	1	1	2	2	2	2
186	2	1	1	1	2	2	1	2
187	2	1	1	1	2	2	1	1
190	2	1	1	1	2	2	2	2
191	2	2	2	1	2	2	1	1
192	2	2	2	1	2	2	1	2
193	2	2	2	1	2	2	1	2
194	2	2	2	1	2	2	1	1
195	2	2	2	1	2	2	2	2
196	2	2	2	1	2	2	2	2
197	2	2	2	1	2	2	1	2
198	2	2	2	1	2	2	2	1
199	2	2	2	1	2	2	1	2
200	2	2	2	1	2	2	2	2
201	2	2	2	1	2	2	1	2
202	2	2	2	1	2	2	1	2
203	2	2	2	1	2	2	1	2
204	2	2	2	1	2	2	1	2
205	2	2	2	1	2	2	1	1
206	2	2	2	1	2	2	2	2
207	2	2	2	1	2	2	1	2
208	2	2	2	1	2	2	2	1
209	2	2		1	2	2	1	2
210	2	2	2	1	2	2	1	2
211	2	2	2	1	2	2	1	2
212	2	2	2	1	2	2	1	1
213	2	2		1	2	1	2	2
214	2	2	2	1	2	2	1	2
215	2	2	2	1	2	2	2	2
216	2	2	2	1	2	2	1	2
217	2	2	2	1	2	2	2	2
218	2	2	2	1	1	2	1	2
219	2	2	2	2	2	2	2	1
220	2	2	1	1	2	2	1	2
221	2	2	2	1	2	1	2	2
222	2	2	2	1	2	2	2	1
223	2	2	2	1	2	2	1	2
224	2	2	2	1	2	2	2	2
225	2	2	2	1	2	2	2	2
226	2	2	2	1	2	2	1	2
227	2	2	2	1	2	2	2	2
228	2	2	2	1	2	2	2	2
229	2	2	2	1	2	2	2	2

230	2	2	2	1	2	2	1	2
231	2	2	2	1	2	2	1	1
232	2	2		1	2	2	1	2
233	2	1	1	4	2	2	2	2
234	2	2	1	1	2	2	2	1
235	2	2	2	1	2	2	1	2
236	2	2	2	1	2	2	2	2
237	2	2		1	2	2	1	1
238	2	2	2	1	2	2	1	1
239	2	2	2	1	2	2	1	2
240	2	2	2	1	2	2	1	1
241	2	2		1	2	2	2	1
242	2	2		1	2	2	1	2
243	2	2	2	1	2	2	2	2
244	2	2	2	1	2	2	2	2
245	2	2		1	2	2	1	1
246	2	2	2	1	2	2	1	2
247	2	2	2	1	2	2	1	1
248	2	2		1	2	2	1	2
249	2	2		1	2	2	1	1
250	2	2	1	2	2	2	1	1
251	2	1	1	3	2	2	1	1
252	2	2	2	2	2	2	2	2
253	2	2	2	1	2	2	1	2
254	2	2	2	1	2	2	2	2
255	2	2	2	1	2	2	1	2
256	2	2	2	1	2	2	2	2
257	2	2	2	1	2	2	2	2
258	2	1	1	3	2	2	2	2
259	2	2	2	1	2	2	1	1
260	2	2		1	2	2	1	2
261	2	2	2	1	2	2	1	1
262	2	2	2	1	2	2	1	1
263	2	2	2	1	2	2	2	2
264	2	2		1	2	2	2	2
265	2	2	2	1	2	2	2	2
266	2	2	2	1	2	2	1	2
267	2	2	2	1	2	2	2	2
268	2	2	1	1	2	2	2	1
269	2	2	2	1	2	2	2	1
270	2	2	1	1	2	2	1	2
271	2	1	1	2	1	2	1	1
272	2	2	2	1	2	2	2	2
273	2	2	2	1	2	2	2	2
274	2	2	2	1	2	2	2	2
275	2	2		1	2	2	2	2
276	2	2	2	1	2	2	2	2

277	2	2	2	1	2	2	2	2
278	2	2	2	1	2	2	2	2
279	2	2	2	1	2	2	2	1
280	2	2	2	1	2	2	1	2
281	2	2	2	1	2	2	2	2
282	2	2	2	1	2	2	2	2
283	2	2	2	1	2	2	1	2
284	2	2	2	1	2	2	2	2
285	2	2	2	1	2	2	2	2
286	2	2	2	1	2	2	1	1
287	2	2	2	1	2	2	2	2
288	2	2	2	1	2	2	2	2
289	2	2	2	1	2	2	2	2
290	2	2	2	1	2	2	1	2
291	2	2	2	1	2	2	2	2
292	2	2	2	1	2	2	2	2
293	2	2	2	1	2	2	2	2
294	2	2	2	1	2	2	2	2
295	2	2	2	1	2	2	2	2
296	2	2	2	1	2	2	2	2
297	2	2	2	1	2	2	2	1
298	2	2	2	1	2	2	2	2
299	2	1	1	2	1	2	1	1
300	2	2	1	1		2	2	2
301	2	2	1	1	2	2	2	2
302	2	1	1	1	2	2	1	1
303	2	2	2	2	2	2	2	2
304	2	2	1	1	2	2	1	2
305	2	2	1	2	2	2	2	2
306	2	1	1	2	1	2	1	1
307	2	2		1	1	1	1	1
308	2	1	1	4	2	2	2	2
309	2	1	1	4	1	1	1	1
310	2	2	1	1	2	2	2	2
311	2	1	1	2	2	2	1	2
312	2	1	1	2	1	2	2	2
313	2	1	1	3	1	2	1	1
314	2	1	1	3	1	2	1	1
315	2	2	1	1	2	2	2	2
316	2	2	1	1	1	2	2	1
317	2		2	2	1	2	2	2
318	2	1	2	2	1	2	1	1
319	2	2	1	1	2	2	2	2
320	2	2	1	2	1	2	2	1
321	2	1	1	2	1	2	1	1
322	2	1	1	1	1	1	1	1
323	2	1	1	1	1	2	1	1

324	2	1	2	1	2	2	1	2
325	2	1	2	1	2	2	2	2
326	2	1	2	1	2	2	2	2
327	2	1	2	1	2	2	1	2
328	2	1	2	1	2	2	1	1
329	2	1	2	4	1	2	2	2
330	2	1	1	1	2	1	2	2
331	2	1	2	1	2	1	2	2
332	2	1	2	1	1	1	1	1
333	2	1	2	1	2	2	1	1
334	2	1	2	1	2	2	1	2
335	2	1	2	1	2	2	1	2
336	2	1	2	1	2	2	2	2
337	2	1	2	1	1	2	2	1
338	2	1	2	1	2	2	2	2
339	2	1	2	1	2	2	2	2
340	2	1	2	1	2	2	2	2
341	2	1	2	1	2	2	2	2
342	2	1	2	1	1	2	1	2
343	2	1	2	1	2	2	2	2
345	2	1	2	1	2	2	1	2
346	2	1	2	1	2	2	1	1
347	2	1	2	4	1	2	2	1
348	2	1	2	1	2	2	2	2
349	2	2	1	1	2	2	2	2
350	2	1	2	2	1	2	1	1
351	2	2	1	1	2	2	2	1
352	2	1	1	3	2	2	2	1
353	2	2	1	1	2	2	1	1
354	2	1	1	2	2	2	2	1
355	2	2	1	1	2	2	2	2
356	2	1	1	3	2	2	2	1
357	2	1	1	1		2	2	1
358	2		2	2	2	2	1	1
359	2	1	1	2	1	2	1	1
360	2	2	1		2	2	2	1
361	2	1	1	1	1	2	2	1
362	2	2	1	1	2	2	2	2
363	2	2	1	2	1	2	1	1
364	2	2	1	1	1	2	2	1
365	2	2	2	1	2	2	2	2
366	2	1	2	1	2	2	2	2
367	2	2	2	2	2	2	2	1
368	2	1	2	1	2	2	1	2
369	2	1		1	2	2	1	2
370	2	1	2	1	2	2	1	1
371	2	1	2	1	2	2	1	2

372	2	1	2	2	1	1	1	2
373	2	1	2	1	2	2	2	2
374	2	1	2	1	2	2	1	2
375	2	1	1	1	2	2	2	1
376	2	2	2	1	2	2	2	1
377	2	2	1	1	2	2	2	2
378	2	2	1	1	2	2	2	2
379	2	2	1	1	2	2	1	2

Q_2d	Q_2e	Q_3a	Q_3b	Q_3c	Q_3d	Q_3d_spec	Q_4	Q_5
2	1	1	2	2	2		2	2
1	2	1	2	2	2		2	2
2	2	1	2	2	2		2	2
2	2	1	2	1	2		2	2
2	2	1	2	2	2		2	2
2	2	1	1	1	2		1	1
2	2	1	2	2	2		2	2
2	2	2	1	2	2		1	2
2	1	2	2	1	2		1	2
2	2	1	2	2	2		1	2
1	2	1	2	2	2		2	2
2	2	1	1	1	2		1	2
2	2	1	2	1	2		2	2
2	1	1	2	1	2	time consu	2	
2	2	1	2	2	2		2	2
2	2	1	2	2	2		2	2
1	2	1	2	2	1	more newe	2	2
1	2	1	1	2	2		1	1
2	1	1	2	1	2		2	1
2	1	1	2	1	2		2	1
2	1	1	2	1	2		2	1
1	2	1	2	1	2	cause crack	2	2
1	2	1	2	1	2	causes crac	2	2
1	2	1	1	1	2		2	2
1	2	1	1	1	2		2	2
2	1	2	2	2	1	retention, (	2	1
2	1	2	2	2	1	retention, (	2	1
2	2	1	2	1	2		2	2
2	2	1	2	1	2		2	2
2	1	1	1	1	2	also becaus	2	1
2	1	1	1	1	2	also becaus	2	1
2	2	1	2	1	2		2	2
2	2	1	2	1	2		2	2
1	2	1	2	2	2		2	2
2	1	1	2	2	2		2	2
2	2	2	2	1	2		2	2
2	2	1	2	1	2		2	2
2	2	1	2	1	2		2	2
2	1	1	1	1	1		1	1
2	2	1	2	1	2	a	2	2
2	1	1	1	1	2		1	1
1	2	1	2	1	2		2	2
1	2	1	2	1	2		2	2
1	2	1	2	1	2		2	2
1	2	1	2	1	2		2	2
2	2	2	1	1	2		1	2

2	2	1	2	1	2		2	2
1	2	1	2	1	2		2	2
2	1	2	2	2	2	not a resto	2	2
1	2	1	2	2	2		2	2
2	2	1	2	2	2		2	2
2	2	1	2	2	2		2	2
2	2	1	2	2	1	cavity conf	2	2
1	2	1	2	1	2		2	2
2	2	1	2	2	2		1	2
2	2	1	2	1	2		2	2
2	2	1	2	2	2		2	2
2	2	2	1	2	2		1	2
2	2	1	2	1	2		2	2
2	2	1	1	1	2	if there is 3	1	
2	2	2	2	1	2		1	2
2	1	1	1	1	2		1	1
2	2	1	2	2	2		2	2
2	2	1	2	2	2		2	2
2	2	2	1	2	2		1	2
1	2	2	2	2	2		2	2
2	1	1	2	2	2		2	2
2	2	2	2	1	2		2	2
2	2	2	2	1	2		2	2
2	2	1	2	2	2		2	2
2	2	1	2	2	2		2	2
2	1	2	2	2	1		2	2
2	1	1	1	1	1		1	2
2	2	1	2	1	2		2	2
2	1	1	2	2	2		2	2
2	1	2	2	2	1	my specialt	2	2
2	2	1	1	1	2	condensati	2	2
2	1	1	2	1	2		2	2
2	2	1	2	1	2		2	2
2	2	1	1	2	2		2	2
2	2	1	1	2	2		2	2
2	2	1	2	1	2	depending	1	2
2	2	1	2	1	2		2	2
2	2	1	2	1	2		2	2
2	2	1	2	1	2	depending	1	2
2	1	1	2	2	2		2	1
2	2	2	2	1	2		2	2
1	2	1	2	1	2		2	1
1	2	1	2	1	2		2	2
2	1	2	2	2	1	preserve tc	2	1
2	1	2	2	1	2		2	2
1	2	1	2	1	2		2	2
1	2	1	1	2	2		1	2

2	2	2	2	1	2		2	2
2	2	1	2	1	2	cavity suita	2	2
1	2	1	2	2	2		2	2
1	2	1	2	1	2		2	2
2	2	1	2	2	2		2	2
2	2	1	2	2	2		2	2
2	1	1	2	2	2		2	1
2	1	1	2	2	2		1	2
1	2	1	2	1	2		2	2
2	2	1	2	1	2		2	2
2	2	2	2	1	2		2	2
2	1	1	2	2	2		2	2
2	1	1	2	2	2		2	2
1	2	1	2	1	2		2	2
1	2	2	2	1	2		2	2
2	1	2	1	2	2		1	1
2	1	2	2	1	2		2	1
2	1	2	2	2	1	im not rest	2	2
2	1	1	2	1	2		2	1
2	2	1	2	2	2		2	2
2	1	1	2	2	2		2	2
1	2	1	2	1	2		2	2
2	1	2	2	1	2		1	1
2	2	1	2	2	2		2	2
2	2	1	2	2	2		1	2
2	2	1	2	1	2		2	2
2	1	1	2	1	2		2	2
2	1	1	2	1	2		2	2
2	2	1	1	1	2		1	2
2	2	1	2	1	2		2	2
2	2	2	2	1	2		2	2
2	2	1	1	2	2		2	2
2	1	1	2	2	2		2	2
2	2	1	2	2	2		1	1
2	2	1	2	2	2		2	2
2	1	1	2	1	2		2	2
2	2	1	2	2	2		1	2
1	2	1	2	1	2		2	2
2	1	1	2	1	2		2	2
2	2	1	1	1	2		1	1
2	2	2	2	1	2		2	2
1	2	1	2	1	2		2	2
2	2	1	2	2	1	small caviti	2	2
1	2	1	2	1	2		2	2
1	2	1	2	2	2		2	2
2	2	2	2	1	2	honestly i p	2	2
2	2	1	1	1	2		1	2

2	1	2	2	2	1	need time	2	2
2	2	1	2	1	2		2	2
1	2	1	2	1	1	bonding	2	2
1	2	1	2	1	2		2	2
1	2	1	2	1	2		2	2
2	2	1	2	2	2		2	1
1	2	1	1	2	2		1	2
2	1	1	1	2	2		1	2
2	2	1	2	1	2		2	2
2	2	2	1	2	2		1	2
2	2	2	1	2	2		1	2
1	2	2	1	1	2		1	2
2	1	2	1	2	2		1	2
2	1	2	1	2	2		1	2
2	1	1	2	2	2		2	2
2	2	1	1	1	2		1	1
2	2	2	2	1	2		1	2
2	1	1	1	2	2		1	1
2	2	1	2	2	2		2	2
2	2	1	2	2	2		2	2
2	2	1	2	2	2		2	2
1	2	1	2	2	2		1	2
1	2	1	1	2	2		1	2
1	2	1	2	2	2		2	1
2	2	2	2	1	2		2	2
2	2	2	2	1	2		1	1
2	2	1	2	2	2		1	1
2	2	1	2	2	2		2	2
2	2	1	2	2	2		2	2
2	2	1	1	1	2		1	2
2	2	1	2	2	2		2	2
2	2	1	2	1	2		2	2
2	1	1	2	1	1	thermal co	2	1
2	2	1	2	1	2		2	2
2	1	1	2	1	1	sensitivity	1	1
2	2	1	2	2	2		1	1
2	2	1	2	1	2		2	2
2	2	2	1	2	2		1	2
2	2	1	1	1	2		1	2
1	2	1	1	1	2		1	2
2	2	1	1	1	2		1	2
1	2	1	1	1	2		1	2
2	1	2	1	1	2		1	1
2	2	1	1	2	2		2	2
2	1	1	1	2	2		1	2
2	1	2	1	1	2		1	1
2	1	1	2	1	2		1	1

2	2	2	1	1	2		1	1
2	2	1	1	2	2		1	2
2	2	1	2	2	2		2	2
1	2	2	2	1	2		2	2
1	2	1	1	1	2		1	2
2	2	1	2	2	2		1	1
2	1	1	2	1	2		1	2
2	2	1	2	2	2		2	2
1	2	1	2	2	2		2	2
2	2	1	2	2	2		2	2
2	2	1	2	2	2		2	2
2	2	2	1	2	2		1	1
2	2	2	2	1	2		1	2
1	2	1	2	1	2		2	2
2	1	1	2	2	2		1	1
2	2	2	1	2	1	time	1	2
2	2	2	2	1	2		2	2
1	2	1	2	1	2		1	2
2	2	1	2	1	2		2	2
2	2	1	2	1	2		2	2
1	2	1	2	2	2		2	2
1	2	1	1	1	2		1	2
1	1	1	1	1	2		1	1
2	2	1	2	2	2		2	2
2	1	2	2	2	1	i hate it	1	2
2	2	1	2	2	2		2	2
2	1	1	2	1	2		1	1
2	1	1	2	1	2		1	1
2	1	1	2	1	1		2	2
2	2	1	2	1	2		1	1
2	2	1	2	2	2		1	2
1	2	1	1	2	2		1	2
1	2	1	1	2	2		1	2
2	1	1	1	1	2		1	2
2	1	1	2	2	2		1	1
2	1	1	2	1	2		2	1
2	2	1	2	2	2		1	2
2	1	1	2	1	2		1	2
2	2	1	1	1	2		1	2
1	2	1	2	1	2		2	2
2	2	1	1	1	2		1	1
2	2	2	2	1	2		2	2
2	1	1	2	1	2		2	1
2	1	1	2	1	2		2	1
2	1	1	2	2	2		2	1
2	1	2	2	1	2		2	1
2	1	1	2	2	2		2	1

2	1	1	2	1	2		2	1
2	1	1	2	1	1		2	1
2	1	1	2	1	2		2	1
2	1	1	2	2	2		2	1
2	1	1	2	2	2		2	1
2	1	1	1	1	2		1	1
2	1	2	2	1	2		1	1
2	1	1	1	1	2		1	1
2	1	1	2	2	2		1	1
2	2	1	2	2	2		2	1
2	1	2	2	1	2		2	1
2	1	1	1	1	2		1	1
2	1	1	2	2	2		2	1
2	2	1	2	2	2		1	1
2	1	1	1	1	2		1	2
2	1	2	2	2	1		2	1
2	1	1	1	1	2		1	2
2	1	1	2	1	2		1	1
2	1	2	2	1	2		1	2
2	1	1	1	1	2		1	2
2	1	1	2	2	2		2	2
2	1	1	2	2	2		1	1
1	2	1	1	1	2		2	2
2	1	1	1	1	2		1	2
2	1	1	1	1	2		1	2
2	2	1	2	2	2		2	2
1	2	1	1	1	2		1	2
2	2	2	2	1	2		1	1
2	1	1	1	1	2		2	1
1	2	1	1	1	2		1	2
1	2	2	2	1	2		2	2
2	1	1	2	1	2		2	2
1	1	2	2	1	2		2	2
2	1	1	1	1	2		1	1
1	2	1	2	1	2		2	2
1	2	1	2	2	2		2	2
1	2	2	2	1	2		1	2
1	2	1	1	1	2		2	1
2	1	1	1	1	2		1	2
1	2	2	2	1	2		2	2
1	2	1	2	1	2		2	1
2	2	1	1	1	2		2	2
2	1	1	1	1	2		1	1
1	2	2	2	1	2		2	2
1	2	1	1	1	2		2	2
1	2	2	2	1	2		1	2
2	2	1	1	1	2		2	2

2	2	2	1	2	2		1	2
2	1	1	2	2	2		1	2
2	1	2	2	1	2		2	1
1	2	2	2	2	1	bad materi	1	2
2	2	2	1	1	2		2	2
1	2	1	2	2	2		2	1
2	2	1	2	1	2		1	1
2	2	2	2	1	2		2	2
2	2	1	2	1	1	economic s	2	2
2	2	2	1	1	2		2	2
2	2	1	2	1	2		2	2
2	2	1	2	1	2		1	2
2	1	1	2	2	2		1	1
2	2	2	2	2	2		1	2
2	1	1	1	2	2		2	2
1	2	1	1	1	2		1	1
2	1	1	1	1	2		2	1
2	1	2	2	1	2		1	1
2	2	2	2	2	1	none	1	2
2	1	1	2	2	2		2	2
2	2	1	2	2	2		1	2
1	2	1	2	1	2		2	2
2	2	1	2	2	2		2	2
2	1	2	2	1	2		2	1
1	2	1	2	2	2			2
1	2	2	2	1	2		2	2
1	2	1	2	1	2		2	2
2	2	2	2	1	2		2	2
1	2	1	1	1	2		2	2
2	2	1	2	1	2		2	2
2	1	1	1	1	2		1	2
1	2	1	1	1	2		2	2
1	2	2	2	1	2		1	1
1	2	1	1	1	2		2	1
1	2	2	2	1	2		2	2
1	2	1	2	1	2		1	1
2	2	2	2	1	2		1	2
2	1	1	1	1	2		1	2
2	2	1	2	1	2		2	2
1	2	1	1	1	2		2	2
2	1	1	2	2	2		1	1
2	1	2	2	2	1		2	1
2	2	2	2	1	2		1	1
2	2	1	2	2	2		2	2
2	2	1	2	2	2		2	2
2	2	1	2	2	2		1	2
2	2	1	2	1	2		1	2

2	2	1	2	2	2			
2	1	1	2	2	2		1	2
1	2	1	2	2	1	difficult ma	2	2
1	2	1	1	1	2		1	2
1	2	1	1	2	2		1	2
1	2	1	2	2	2		1	1
1	2	1	2	2	2		1	1
2	2	1	2	2	2		1	1

Q_6	Q_7	Comments	Job_czat
1	2	some clinic	1
2	2	isolation	1
	2	can't be rep	2
1	2	Arabic	2
1	2		1
1	1	arabic	1
2	2	arabic	1
1	2		1
2	2	arabic	2
1	1	toxicity of r	2
2	2		2
1	2	arabic	1
2	2	arabic	1
1	2	advantages	1
1	2	arabic	1
	2	amalgam h	1
	2	arabic	1
1	1	amalgam ta	1
1	1	arabic	1
1	1		1
1	1		1
1	2	no scientifi	2
1	2	no scientifi	2
1	1	arabic	2
1	1	arabic	2
1	2	no justifiab	1
1	2	no justifiab	1
1	2		2
1	2		2
	2	arabic	2
	2	arabic	2
1	2	arabic	2
1	2	arabic	2
1	2	arabic	2
1	2	arabic	2
1	2	arabic	1
3	2	bad oral hy	1
1	2	arabic	1
1	1	a	1
1	2	a	1
1	1	a	2
1	2	a	2
1	2	a	2
	2	safe materi	2
	2	safe materi	2
1	2	a	1

2	2	a	1
1	2	good longe	1
1	2	safe	2
1	2	a	2
1	2	a	2
2	2		2
1	2	there are c	1
1	2	a	1
1	2	a	1
1	2	not all case	1
1	1	a	1
1	1	a	1
2	1	a	1
3	2	a	2
1	2	a	1
1	1	a	2
2	2	the absenc	1
2	2	the absenc	1
2	2	a	2
	2	a	2
1	2		1
1			1
1	2	composite	2
2	2	a	1
1	2	because sti	2
2	2	a	1
1	2	a	1
1	2	a	1
2	2	a	1
1	2	a	2
	1	patient des	2
1	1	a	1
	2		1
1	2	very large c	1
1	2	very large c	1
1	2	a	1
2	2	a	2
2	2	a	2
1	2	a	1
1	1	a	1
2	2		1
2	2	a	1
2	2	a	1
1	1	a	1
1	1	a	1
1	2	a	1
3	2	because it l	2

1	2	a	1
2	2	no approve	1
1	2	uncontrolo	1
1	2	because its	1
1	2	toxicity not	2
	2	i am perioc	2
1	1		1
1	2	depends or	2
3	2	a	2
1	2	a	2
1	2		1
1	1	a	1
1	1	a	2
2	2		1
2	2	a	1
1	1		1
1	2		2
1	2	a	1
1	1		1
2	2	because nc	1
1	2		1
1	2	we need it	1
1	1	a	1
1	2		2
1	2	a	1
2	2		2
1	2		1
1	2		1
1	2	amalgam g	2
	2	a	1
2	2		1
1	2	a	1
1	2	a	1
1	1	because co	1
2	1	a	1
1	2	high succes	1
2	2	a	1
1	2	a	1
1	1		1
1	1	mercury to	1
1	2	it has great	1
1	2	a	1
2	2	a	1
1	2	a	1
1	2	a	1
1	2	amalgam is	1
1	2		1

2	2	more longe	1
2	2	best in long	1
1	2	a	1
1	2	a	1
1	2		1
1	1	esthetic	1
1	1		1
1	1	a	1
1	2	a	1
2	2	because of	1
2	2	because lar	1
1	2	amalgam n	1
1	1	a	1
1	1	a	1
1	1	esthetics	1
1	1	not estheti	1
1	1		1
1	1		1
1	2	a	1
1	2	a	1
1	2	a	1
1	1		1
1	1		1
1	2	a	1
1	2	a	1
1	1	a	1
1	1		1
1	2		1
1	1	a	1
2	2		1
2	2	their streng	1
1	2		1
1	1		1
2	2	a	1
1	1		1
1	2	because its	1
1	1		1
1	2	a	1
1	1	a	1
1	2	a	1
1	2		1
2	1	a	1
1	2	a	1
1	2		1
1	1		1
1	1	esthetics ar	1
1	1		1

1	2	the need fc	1
2	1		1
1	1		1
1	1	esthetics is	2
1	1		1
1	2		1
1	2	deep caviti	1
1	2		1
1	1		1
1	1		1
1	2	a	1
1	1		1
1	2	strength m	1
1	2	core mater	1
1	1		1
1	2	composite	1
1	1	mercury to	1
1	2		1
1	1		1
1	2		1
1	1		1
1	2		2
3	1		1
1	2		1
1	1	a	1
1	1		1
1	1		1
1	1		1
1	1	a	2
1	1		1
1	2	a	1
1	2	amalgam h	1
1	2	a	1
1	1	toxicity anc	1
1	1		1
1	1		1
1	1		1
1	2	pedo patie	1
1	1		1
1	1		1
1	1		1
2	2		1
1	1		1
1	1		1
1	1		1
1	1		1
1	1		1

1	1		1
1	1		1
1	1		1
1	1		1
1	1	a	1
2	1		1
1	1		1
1	1	a	1
1	1		1
1	1		1
1	1		1
1	1		1
1	1		1
1	1		1
1	1		1
1	1		1
1	1		1
1	1		1
1	2	a	1
1	1		1
1	1		1
1	1		1
1	1		1
1	1		1
1	1		1
1	2		1
1	1		1
1	2	a	1
1	1		1
1	1		1
1	2		1
1	1	composite	2
2	2	durable am	2
1	1	nobody like	1
1	1	new compo	1
1	1		1
1	2		2
1	1	composite	2
1	1	amalgam is	1
2	2		1
1	1		1
2	2		1
1	1		1
1	1		1
2	2		1
2	2		1
3	2		1

1	2	a	1
1	2	a	1
1	2	a	1
1	2	a	1
1	2	large restor	1
1	2		2
1	1	esthetic	1
1	2		1
1	1	a	1
1	2	a	1
1	2	isolation	1
1	1	a	1
1	1	not estheti	1
1	1		1
1	1		1
1	2	a	1
1	2	a	1
1	1	a	1
2	2	a	1
1	2	a	1
1	2		1
2	2		1
1	2	a	2
1	2	a	1
1	1		1
2	2		1
1	1		1
1	2		2
1	1		1
1	2		1
1	2		1
1	1		2
1	1		1
1	1		1
2	2		1
1	2		
1	2		1
1	1		1
1	1		1
1	2		1
1	1		1
1	1		1
1	2	a	1
1	2		1
1	2		1
1	2	a	1
1	2	a	1

		1
1	2 a	1
1	2 a	1
1	1	1
1	1	1
1	1	1
1	1	1
1	1 a	1
